# Neurons within the trigeminal mesencephalic nucleus encode for the kinematic parameters of the whisker pad macrovibrissae

**DOI:** 10.14814/phy2.13206

**Published:** 2017-05-26

**Authors:** Ombretta Mameli, Marcello A. Caria, Francesca Biagi, Marco Zedda, Vittorio Farina

**Affiliations:** ^1^ Department Clinical and Experimental Medicine: Human Physiology Division Sassari Italy; ^2^ Department Veterinary Medicine: Anatomy of Domestic Animals Division Sassari Italy

**Keywords:** Me5 proprioception of macrovibrissae movements, Me5 whisking‐neurons, rat, trigeminal mesencephalic nucleus

## Abstract

It has been recently shown in rats that spontaneous movements of whisker pad macrovibrissae elicited evoked responses in the trigeminal mesencephalic nucleus (Me5). In the present study, electrophysiological and neuroanatomical experiments were performed in anesthetized rats to evaluate whether, besides the whisker displacement per se, the Me5 neurons are also involved in encoding the kinematic properties of macrovibrissae movements, and also whether, as reported for the trigeminal ganglion, even within the Me5 nucleus exists a neuroanatomical representation of the whisker pad macrovibrissae. Extracellular electrical activity of single Me5 neurons was recorded before, during, and after mechanical deflection of the ipsilateral whisker pad macrovibrissae in different directions, and with different velocities and amplitudes. In several groups of animals, single or multiple injections of the tracer Dil were performed into the whisker pad of one side, in close proximity to the vibrissae follicles, in order to label the peripheral terminals of the Me5 neurons innervating the macrovibrissae (whisking‐neurons), and therefore, the respective perikaria within the nucleus. Results showed that: (1) the whisker pad macrovibrissae were represented in the medial‐caudal part of the Me5 nucleus by a single cluster of cells whose number seemed to match that of the macrovibrissae; (2) macrovibrissae mechanical deflection elicited significant responses in the Me5 whisking‐neurons, which were related to the direction, amplitude, and frequency of the applied deflection. The specific functional role of Me5 neurons involved in encoding proprioceptive information arising from the macrovibrissae movements is discussed within the framework of the whole trigeminal nuclei activities.

## Introduction

Among the open questions regarding the rodent whisker‐dependent behaviors, an important aspect is undoubtedly constituted by the way the rat brain processes signals generated by active movements of the macrovibrissae to reconstruct a real image of the surroundings.

It is known that the rodent vibrissae system is formed by two different sensory detectors: the long and short whiskers. A further functional classification distinguishes the laterally oriented long “macrovibrissae,” considered to be a distance‐detecting/object‐locating sense organ, and the shorter, more numerous and more frontal “microvibrissae” which are instead considered to be an object‐recognizing sense organ (Brecht et al. 1997). The microvibrissae numerousness provides in fact a sampling efficacy that is crucial for tactile object recognition while plays a negligible role in detecting spatial information.

With regard to the architecture of the whisker sensory system, the macrovibrissae are embedded in a special pad on both sides of the animal muzzle, aligned in five horizontal regular rows designed as A to E. In each row, the macrovibrissae are arranged almost perpendicular to the animal rostral‐caudal axis, with a specific dorsal‐ventral orientation and a length that significantly increases in the rostral‐caudal direction. Moreover, each vibrissa is aligned with the correspondent of the adjacent rows so that they are also arranged along a series of vertical arcs oriented in the dorsal‐ventral direction. Each vibrissa can be therefore identified by a letter that indicates its row and a number that indicates the arc to which it belongs. Such regular arrangement has been interpreted as a functional architecture in which each whisker appears to act as a lever‐like transducer that provides information as to whether or not, but not where, a single vibrissa had been deflected. The mystacial macrovibrissae have been therefore considered as a distance‐detector array able to acquire head centered spatial information at various dorsal‐ventral angles represented by the disposition of the vibrissae rows (Brecht et al. 1997). Contact between whiskers and objects produces time‐varying stresses at the base of the macrovibrissae (Birdwell et al. 2007) that are transduced into action potentials by the follicles mechanoreceptors (Zucker and Welker 1969; Dőrfl 1982; Gibson and Welker 1983; Szwed et al. 2003, 2006; Stüttgen and Schwarz 2008) and then relayed to the CNS by the trigeminal nerve.

It has long been accepted that lateral stresses applied to the whiskers excite the trigeminal ganglia (TG) neuron terminals in a direction and velocity‐dependent fashion (Zucker and Welker 1969; Gibson and Welker 1983; Lichtenstein et al. 1990; Szwed et al. 2003, 2006). However, we believe that this functional hypothesis must be reconsidered in view of the recent studies indicating that our understanding of the way these responses are processed within the trigeminal system needs to incorporate a new role played by the mesencephalic nucleus (Me5). It has been demonstrated in fact that spontaneous and artificial movements of the macrovibrissae, without touching any object, elicited significant responses in the rat Me5 nucleus (Mameli et al. 2010, 2014). Moreover, neuroanatomical procedures showed that a retrograde tracer injected into the mystacial pad extensively labeled a discrete number of Me5 neurons, while the injection of an anterograde tracer into the Me5 nucleus demonstrated that the peripheral terminals of these same neurons targeted the upper part of the macrovibrissae with fibers spiraling around the circumference of the vibrissae shaft (Mameli et al. 2014). Altogether, for the first time, these studies demonstrated that besides the TG, even the Me5 neurons are involved in macrovibrissae innervation, which role we speculated to be related with encoding spatial information relative to vibrissae movements (Mameli et al. 2010, 2014).

With regard to the macrovibrissae sensory afference to the trigeminal ganglia, it is known that each TG neuron receives inputs from a single whisker and that the receptive fields of these neurons are loosely arranged in a somatotopic fashion (Kerr and Lysak 1964; Zucker and Welker 1969; Erzurumlu and Killackey 1983; Leiser and Moxon 2006). Sensory inputs arising from TG are relayed and somatotopically mapped at each level of the central trigeminal pathways as a spatially orderly sets of neuronal modules (Zucker and Welker 1969). These modules are called “barrelettes” in the brainstem trigeminal nuclei, “barreloids” in the thalamic ventral‐posterior‐medial (VPM) nuclei and “barrels” in the neocortex (Woolsey and Van der Loos 1970; Van der Loos 1976; Ma and Woolsey 1984; Ma 1991; Erzurumlu et al. 2010).

Main aim of the present study was to evaluate whether the Me5 neurons, besides being activated by spontaneous and artificial movements of the macrovibrissae are also involved in encoding specific kinematic parameters of their movements. A further aim was to analyze whether, as reported for the trigeminal ganglion (Brecht et al. 1997), even within the Me5 nucleus exists a neuroanatomical representation of the whisker pad macrovibrissae.

## Materials and Methods

The experiments were performed on thirty young male Wistar rats 250–350 g b.w. (Charles River, Calco, Lecco, Italia s.r.l.), which were maintained under controlled conditions of temperature (23 ± 1°C) and lighting (lights on 7.00–19.00 h), in accordance with the European Communities Council Directive of November 24, 1986 (86/609 EEC) and the EU Directive 2010/63/EU for animal experiments. Laboratory chow diet and water were available ad libitum, and experimental procedures were performed during daytime, taking adequate care to minimize pain or discomfort.

Prior approval for the use of laboratory animals and all the procedures adopted in these experiments was obtained from the Italian Health Ministry and the local Veterinary Health Service (CIBASA). All efforts were made to minimize the number of animals used and their suffering.

The animals were subdivided into two groups, which were submitted to electrophysiological or neuroanatomical experiments respectively.

### Electrophysiological procedures (Group 1)

The goal of this part of the study was to investigate whether the Me5 neurons, which, as previously demonstrated responded to spontaneous and artificial movements of the vibrissae (Mameli et al. 2010, 2014), are also involved in encoding the related kinematic parameters and specifically the direction, frequency, and amplitude of single displacements.

The animals (*n* = 12) were anesthetized by intraperitoneal injection of diazepam (30 mg/kg) and ketamine hydrochloride (45 mg/kg) and then mounted prone in a stereotaxic frame (David Kopf, Instruments, Tujunga, CA). A craniotomy was performed at occipital bone level to expose the cerebellum and the obex. All the exposed surfaces were then protected with warm mineral oil and paraffin (37°C) and every 40 min, the pressure points were injected with xylocaine (0.3%). The ECG was continuously monitored to assess the anesthesia level and prevent the animal discomfort.

At the end of surgical procedures a tiny iron rod was fixed to the mandibular symphysis with dental acrylic cement and then connected to an automatic custom‐made stimulating device (capacitor powered by an AD 9 V battery). This set up prevented orofacial movements related to breathing and/or spontaneous chawing activities. Furthermore, when switched on, the device delivered a standard pulse to a magnet, which pulled in turn the tiny iron rod sealed to the mandibular symphysis thus eliciting a standard stretch of the jaw‐closing muscles (Poliakov and Miles 1994). This stretch‐test was used to identify the correct location of the recording site within the Me5 nucleus. It is in fact known that the mesencephalic neurons innervate the muscle spindles of jaw‐closing muscles and mediate the jaw jerk or mandibular stretch reflex (McIntyre 1974).

The whisker pad macrovibrissae were then inserted, using a delicate hook, into the holes (2.5 × 2.5 mm each) of a very light and inextensible grid (1.5 × 1.5 cm; 1.5 mg weigh) positioned parallel to the pad skin and at a minimal distance to avoid any possible contact with the whisker pad even during maximal macrovibrissae deflection. We chose to test the major macrovibrissae, identified as straddlers (*α*, ß, *γ*,* δ*) and A_1_–A_4_, B_1_–B_4_, C_1_–C_4_, D_1_–D_4_, E_1_–E_4_ of the rows A–E (Brecht et al. 1997) because they approximately lie in the same sensory plane and therefore this special planar array allowed the onset of comparable inputs in response to identical mechanical forces exerted by the stimulus. To perform the simultaneous deflection of these macrovibrissae, the grid was connected to an electronic microdrive (David Kopf micropositioner mod. 660) by means of a special custom‐made adapter. Four standardized directions: forward/backward, backward/forward, up/down and down/up were chosen. Each direction was tested using the same velocity (*V* = 2 mm/sec) and amplitude angle (45°) of macrovibrissae deflection. These parameters were chosen since they approximately represented the mean of the whole range of the tested values for speed and amplitude.

The electrophysiological recordings were carried out at the following stereotaxic coordinates: −9.68 to −10.04 mm posterior to the bregma, and 1.2–1.4 mm from the midline (Paxinos and Watson 1997)as previous experiments showed that within these coordinates were located the Me5 neurons responsive to macrovibrissae movements (Mameli et al. 2010, 2014). The electrical activity of the Me5 neurons innervating the whisker pad macrovibrissae (Me5 whisking‐neurons) was extracellularly recorded using tungsten‐in‐glass microelectrodes (impedance: 700–1200 KΩ) slowly advanced into the Me5 nucleus using an electronic microdrive (David Kopf Instruments). In a previous study, it has been shown that the large Me5 neurons innervating the masseter muscle spindles are intermingled with the whisking‐neurons localized in the medial‐caudal part of the Me5 nucleus (Mameli et al. 2010). Therefore, to make sure of the recording site location within the nucleus, at each recording site Me5 nucleus was tested for response to the masseter muscle stretch (Figs. 3A, 5A). The electrical signals were relayed to conventional preamplifiers and then fed to computers for A/D conversion and subsequent analysis (Tecfen computerscope analysis ISC‐16 software, and Power Lab 4/30 Chart 5, V 5.4.2 software). Recordings of whisking‐neurons firing were performed in resting conditions (i.e. vibrissae motionless) and were protracted during and after the macrovibrissae deflection in the standardized directions. A peri‐stimulus‐time‐histogram (PSTH) was constructed to identify the pattern of the responses elicited by the stimulus application.

The electronic microdrive (David Kopf Instruments) that allowed macrovibrissae deflection could be set in different modes. The mode “run” was set to produce a continuous inclination at velocities that ranged from 2 *μ*m/sec up to 4 mm/sec. Different velocities were tested in order to indirectly evaluate whether the Me5 neurons were able to detect also the frequency of macrovibrissae deflection. Moreover, the cumulative frequency distribution (CFD) of the neuron response, obtained by adding up each frequency value to all of the preceding values, was constructed at the tested velocities to evaluate the relative effect.

The mode “burst” was instead set to induce the macrovibrissae deflection in steps, to test the Me5 neuron response at different inclination angles. In particular, the speed of deflections could be changed from 2 *μ*m to 4 mm/sec and the burst amplitude from 1.0 to 99.5 *μ*m to induce various angles of macrovibrissae deflection. At each step, the degree of the macrovibrissae inclination was measured at the end of deflection. Responsive neurons were tested at the following angles: 25, 35, 45, and 70° and to verify the presence of adaptation phenomenon each deflection was kept steady for about 20 sec.

In all tests, the resting activity of the Me5 neurons was recorded following the recovery of macrovibrissae setting into the grid. The Me5 nucleus was then identified by the masseter muscle stretch‐test, and 3 min after recovery, the spontaneous electrical activity of the Me5 neurons were recorded and continuously monitored before, during, and after macrovibrissae deflection.

#### Mapping the electrophysiological recordings

To identify the arrangement of responsive neurons within the Me5 nucleus, its whole medial‐caudal part was probed by performing recordings at different depths and coordinates (−9.68 to −10.04 mm posterior to the bregma and 1.2–1.4 mm from the midline). The stereotaxic coordinates of each recording site were collected and successively used for the tridimensional reconstruction of the recorded Me5 neurons location within the nucleus. The whisking‐neurons responses to macrovibrissae deflection were then coupled to the effective direction. In particular, if their response were elicited by two or more directions, the criteria set to identify the preferential direction were the latency and/or the presence of the adaptation phenomenon. On the contrary, two or more directions were attributed to the recorded unit when comparison among responses did not show any clear difference.

At the end of the experiment, the recording site was marked by an electrolytic lesion (20 *μ*A cathodal current, 20 sec) and the animal sacrificed with an overdose of the anesthetic drugs. The brain was removed, fixed in Carnoy's solution, and embedded in paraffin for subsequent histological procedures. Serial sections (40 *μ*m thick) of the brainstem were cut and stained with cresyl violet (Nissl staining) to verify the position of the last recording site. The correspondent stereotaxic coordinates were used to reconstruct the approximate position of all tested neurons within the nucleus.

### Neuroanatomical procedures (Group 2)

To test the hypothesis of a neuroanatomical arrangement of the Me5 whisking‐neurons within the nucleus, eighteen animals were submitted to a tract‐tracing study. To this purpose, they were first anesthetized with an intraperitoneal injection of diazepam (30 mg/kg) and ketamine hydrochloride (45 mg/kg) and then, under aseptic conditions, a 5% solution of the tracer Dil (1,1′‐dioctadecyl 3,3,3′,3′‐tetramethyl‐indocarbocyanine perchlorate, Molecular Probes, Eugene, OR) was unilaterally injected into the whisker pad using a Hamilton micro‐syringe (1 *μ*L) at a positive pressure (50 *μ*L/min). This tracer was chosen because it is specifically used to label nerve fibers (Tamamaki 1997; Woodhams and Terashima 2000) and axon terminals (Honig and Hume 1989; Berthoud et al. 1992) and also because we have successfully used it in previous studies regarding the macrovibrissae system (Mameli et al. 2008, 2009, 2010, 2014, 2016).

The animals were then subdivided into four subgroups, which were submitted to the tracer injection in specific sites of the whisker pad as follows:

#### Subgroup 2.1 (*n* = 3)

**Figure 1:**
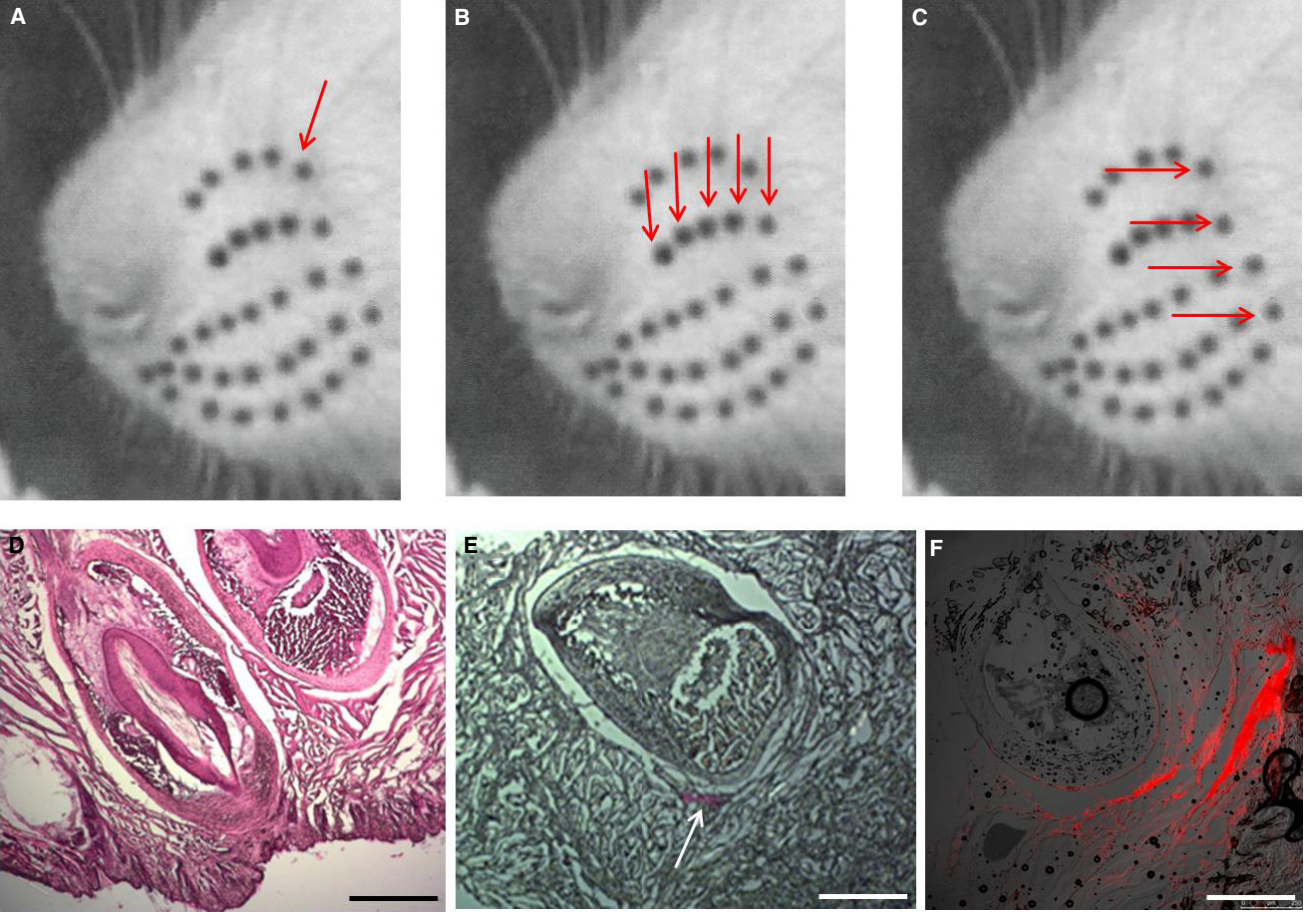
Injection sites of the tracer DIL into the whisker pad in animals of Group 2. (A–C) Rat muzzle showing the macrovibrissae arrangement in the whisker pad (black spots). The arrows indicate the tracer injection sites in animals of subgroups 2.1, 2.2, and 2.3 respectively. (D) Histological hematoxylin‐eosin stained section of the rat follicle‐sinus complexes. (E) Histological section showing the diffusion of the tracer (white arrow) injected in animals of subgroup 2.1, in which one follicle‐sinus complex was labeled. (F) Fluorescence detection of the tracer injected in animals of subgroups 2.2 and 2.3. This figure shows an example of the labeling extent.

In these animals was performed only one injection of the tracer Dil (0.3 *μ*L) in close proximity to a single macrovibrissae of the left whisker pad (Fig. 1A) with the intent of identifying, through retrograde labeling, the correspondent Me5 neuron that provided its innervation. The injection was limited to the perifollicular area, taking great care to spare the integrity of the macrovibrissae follicle external wall and therefore preserve the integrity of the axon terminals within the follicle‐sinus complexes (Ebara et al. 2002). Figure 1E shows the extent of the tracer diffusion around the follicle‐conical body complexes.

#### Subgroups 2.2–2.3 (*n* = 6)

In subgroup 2.2 (*n* = 3), five macrovibrissae of the left‐hand side along a single row were injected following the same procedure as for subgroup 2.1. The vibrissae ß and those of row B_1_–B_4_ were in particular chosen (Fig. 1B). In subgroup 2.3 (*n* = 3), at the same side were injected the straddle whiskers (first arc), i.e. the vibrissae *α*, ß, *γ*,* δ* (Fig. 1C). Figure 1F shows an example of the tracer diffusion in these subgroups.

#### Subgroup 2.4 (*n* = 9)

In these animals, the whole whisker pad was unilaterally and uniformly injected by performing five injections of the tracer (0.05 *μ*L each) to purposely mark all follicle‐conical body complexes of the macrovibrissae.

#### Common procedures

**Figure 2:**
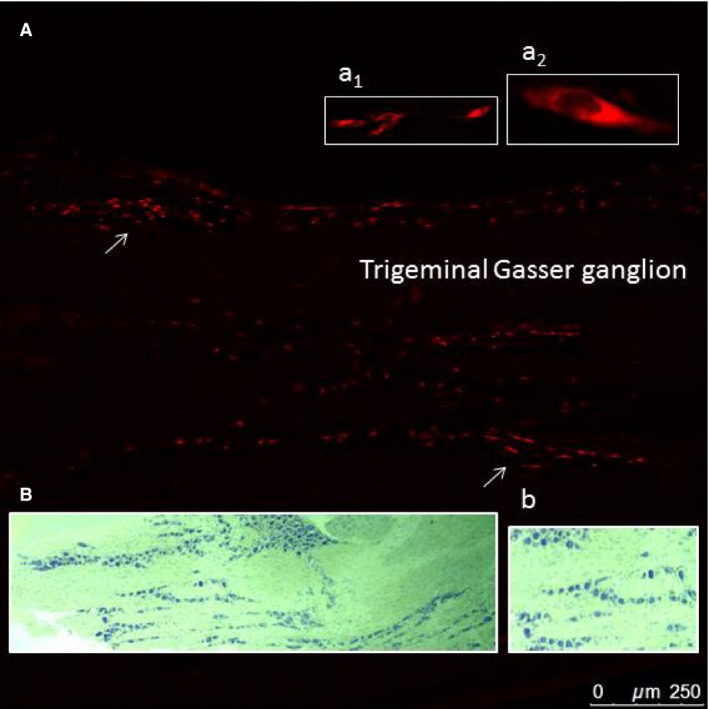
Positive control of the retrograde diffusion of the tracer. (A) Fluorescence detection of a trigeminal Gasser ganglion (GG) longitudinal section labeled by the tracer Dil injected into the ipsilateral whisker pad (from an animal of subgroup 2.4). Arrows indicate some labeled neurons and the insets a_1_ and a_2_ their zoomed views. (B) Histological longitudinal section of the same ganglion stained with cresyl violet showing its structure and (b) a zoomed view of its neurons.

After 7 days, animals of all subgroups were deeply anesthetized through an intraperitoneal injection of diazepam (30 mg/kg) and ketamine hydrochloride (45 mg/kg) and transcardially perfused, throughout the ascending aorta, using 100 mL of saline solution followed by 300 mL of ice‐cold 4% paraformaldehyde in 0.1 mol/L phosphate‐buffered saline solution (PBS, pH 7.4). The brain was removed, post‐fixed for 3–4 h with the same perfusion fixative and cryoprotected overnight in 30% sucrose PBS solution. Then, the brainstem was frozen‐sectioned along the sagittal plane (20 *μ*m thick) using a cryotome. Moreover, in the group 2.4, the brainstem was frozen‐sectioned along the sagittal (*n* = 5 animals) and the coronal planes (*n* = 4 animals). Alternate serial sections were collected in PBS, mounted on glycerin‐albumin‐coated slides and observed using a Leica DMI 6000B microscope, connected to a TCS SP5 Confocal Scanning System (Wetzlar, Germany), with a selective filter for rhodamine (560 nm wavelength). The Me5 nuclei were analyzed to detect the Me5 neurons retrogradely labeled by Dil. As a positive control of the retrograde diffusion of the tracer Dil, five trigeminal Gasser ganglia (TG) from animals of subgroup 2.4 were removed and submitted to the same histological procedures to detect the TG neurons retrogradely labeled by Dil (Fig. 2).

A second series of sections was stained with cresyl violet (Nissl staining) for the histological identification of the neural structures.

In each animal, the pad injected with Dil was removed and frozen‐sectioned to detect the extent of the tracer diffusion around the follicle‐conical body complexes. Alternate serial sections (30 *μ*m thick) were collected in PBS and mounted on glycerin‐albumin‐coated slides for fluorescence detection.

The second series of the sections was stained with hematoxylin‐eosin for histological structures identification. Images of the most significant sections were captured using a Zeiss Axiophot Light Microscope (Oberkochen, Germany).

### Statistical analysis

Changes in the Me5 neurons electrical activity recorded during mechanical deflection of macrovibrissae were statistically evaluated in comparison with the respective spontaneous firing in basal conditions (i.e. macrovibrissae motionless), using the Student's *t*‐test for paired observations.

## Results

### Electrophysiological findings (Group 1)

Results showed that the mechanical deflection of the whisker pad macrovibrissae elicited different patterns of response in the Me5 neurons localized in the medial‐caudal part of the nucleus. To identify spatially ordered sets of neuronal modules in precise areas of the Me5 nucleus, different tracks were performed, several sites tested and the electrical activity of single Me5 whisking‐neurons was recorded in resting conditions, during macrovibrissae deflection and after the end of stimulus application.

Forty two Me5 neurons responsive to macrovibrissae deflection were found in the medial‐caudal area of the nucleus, and the time course of their responses to macrovibrissae deflection analyzed.

**Figure 3:**
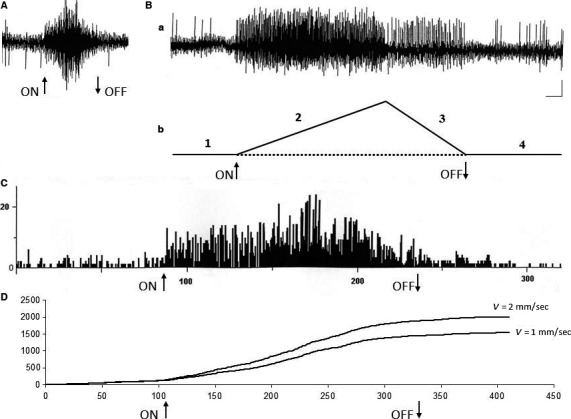
Electrical activity of a Me5 neuron and its response to macrovibrissae deflection in backward/forward direction. (A) Functional identification of the neuron location into the Me5 nucleus elicited by masseter muscle stretch‐test (see method). (B (a)) the Me5 neuron electrical activity recorded in basal conditions, during macrovibrissae deflection in backward/forward direction (mode run, *V* = 2 mm/sec, 45°), during its return to resting position and after the end of the deflection; (b) schematic drawing of the time course of the four tested phases, i.e.: basal conditions (1), macrovibrissae deflection (2), return to the resting position (3) and after the end of the deflection (4). (C) Peri‐Stimulus‐Time‐Histogram (PSTH) of the same neuron activity before, during, and after mechanical deflection of the macrovibrissae in backward/forward direction (mode run, *V* = 2 mm/sec, 45°). (D) Cumulative Frequency Distribution (CFD) of the same neuron constructed from its firing before, during, and after macrovibrissae deflection using the mode run at different velocities. The CFD diagram was obtained adding each frequency value to all of the previous values. In all recordings arrows (ON) and (OFF) indicate the beginning and the end of stimulus application. Calibrations: Horizontal 0.5 sec for A–B, 4 sec/interval for PSTH in C, 50 intervals/division (62.43 ms/bin) for CFD in D; Vertical 0.5 mV for A–B, 10 counts/division for PSTH in C, 500 counts/division for both CFDs in D.

The peri‐stimulus‐time‐histograms (PSTHs) of the Me5 whisking‐neurons firing, constructed before, during, and after the macrovibrissae deflection‐test showed that the mechanical displacement induced a significant short latency increase in the neurons basal firing rate, which was frequently accompanied by a recruitment of other previously silent units. When macrovibrissae went back to their resting position the neurons firing gradually returned to its basal value. Results from all of responsive neurons showed that their firing could increase up to seven times in comparison to basal values. Figure 3B–C shows an example.

With regard to the pattern of their responses to macrovibrissae deflection in the four tested directions, results showed that 80% of the whisking‐neurons responded to the backward‐forward direction being the other directions ineffective or else able to elicit responses at longer latencies.

**Figure 4:**
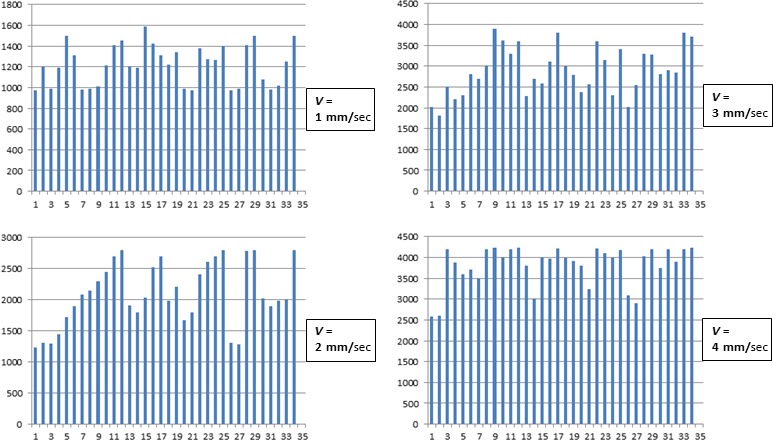
Histograms summarizing the effect elicited in all tested neurons by macrovibrissae deflection in backward‐forward direction at different velocities. Each column refers to a single neuron and indicates the total counts of its firing obtained from the respective CFD (calculated as for Fig. 3) constructed at 62.43 ms/bin during 8 seconds recordings, and with macrovibrissae deflection at 45° angle in backward‐forward direction. Me5 neuron responses were tested at increasing velocities (1–4 mm/sec). Horizontal axes: Neurons analyzed (*n* = 34); Vertical axes: Total counts for each recorded neuron.

Furthermore, the CFD analysis of these responses showed that the Me5 whisking‐neurons were also sensitive to changes in the velocity of macrovibrissae displacement. The mechanical distortion of the Me5 neurons peripheral terminal at macrovibrissae level elicited in fact a significant increase of the unit firing rate in direct correlation with the stimulus velocity, without any change in the pattern of their responses. Figure 3D shows an example. In the range between 2 *μ*m to 4 mm/sec of deflection velocity the Me5 whisking‐neurons significantly increased (*P* < 0.01) their firing rate from 0.42 ± 0.10 Hz (mean ± SD) up to 6.54 ± 0.82 Hz (mean ± SD). To better understand the effects induced by changes of the stimulus velocity, were selected 34 neurons, all responding to the same direction (backward‐forward) during macrovibrissae deflection at a 45° inclination angle. The CFDs of their responses, constructed at 62.43 ms/bin from 8 sec recordings, were carried out and repeated at increased velocities from 1 to 4 mm/sec. The histograms in Figure 4 summarize the trend of the total counts (CFD) detected for each neuron during macrovibrissae deflections performed at different velocities.

**Figure 5:**
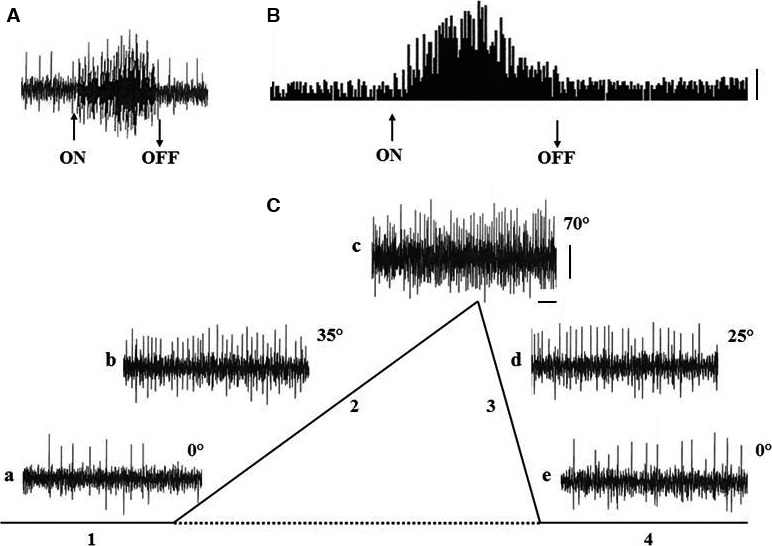
Electrical activity of a Me5 neuron during macrovibrissae deflection performed at different inclination angles. (A) Functional identification of the neuron location into the Me5 nucleus by the masseter muscle stretch‐test. Calibrations, (ON) and (OFF) as for Fig. 3A. (B) Peri‐stimulus‐time‐histogram (PSTH) of the Me5 neuron activity before, during, and after mechanical deflection of the macrovibrissae in backward/forward direction (mode run, *V* = 2 mm/sec, 45°).ON‐OFF interval: 30 sec; Vertical calibration: 10 counts. (C) Recordings of the spontaneous firing of the same neuron, performed at different inclination angles of macrovibrissae deflection (mode burst, *V*: 600 *μ*m/sec) from 0° up to 70°: (a) basal conditions, i.e. at 0°, (b) at 35°, and (c) at 70°. Trace d shows the neuron activity at 25° during macrovibrissae return to their resting position, and trace (e) at 0°. Traces show the peak of neuron firing at precise angles. For each step, the macrovibrissae deflection was maintained steady for about 20 sec to verify the presence of the adaptation phenomenon. The schematic drawing refers to the time course of the tested phases (1–4). Calibrations: Horizontal 1 sec, Vertical 1 mV.

**Figure 6:**
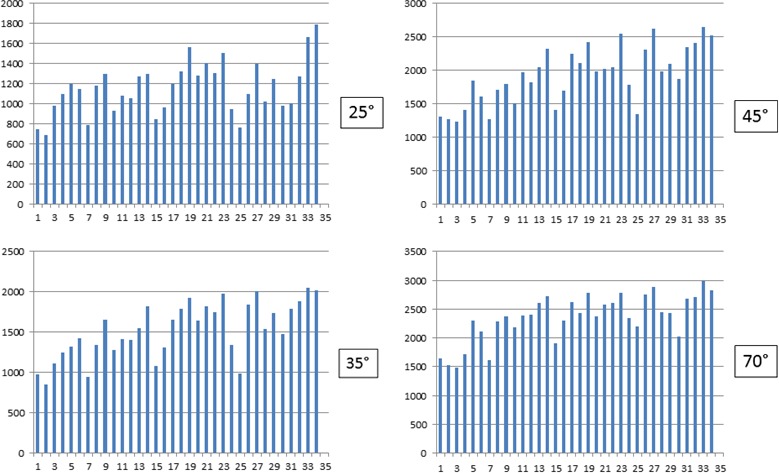
Histograms summarizing the effects elicited in the Me5 neurons by macrovibrissae deflection performed at constant velocity in backward‐forward direction, with different inclination angles. In all histograms, each column refers to a single neuron and indicates the total counts of its firing obtained from the respective CFD (calculated as for Fig. 3) constructed at 62.43 ms/bin and 8 sec analysis, during macrovibrissae deflection at different angles (25–70°) and performed at the constant velocity of 650 *μ*m/sec in backward‐forward direction. Horizontal axes: The neurons analyzed (*n* = 34); Vertical axes: The total counts for each recorded neuron.

As for the capability of the Me5 neurons to detect the amplitude of macrovibrissae deflection, the results showed that the recorded units always increased their firing rate in direct correlation with the amplitude of the mechanical distortion applied, without showing adaptation. Figure 5 shows an example. In particular, during a deflection from 0 to 70°, the whisking‐neurons significantly increased (*P* < 0.01) their spontaneous activity (mean ± SD) from 0.63 ± 0.12 Hz up to a peak of 4.59 ± 0.82 Hz, with a mean value of 0.057 ± 0.002 Hz/sec/degree. The same neurons (*n* = 34), which responded to the backward‐forward direction during macrovibrissae deflection performed at different velocities, were also tested changing the stimulus amplitude but keeping constant its velocity (650 *μ*m/sec). The CFDs of their responses were constructed at 62.43 ms/bin from 8 sec recordings during macrovibrissae deflection carried out at different inclination angles ranging from 25 to 70°. The histograms in Figure 6 summarize the trend of the total counts (CFD) detected in each neuron during macrovibrissae deflections at different inclination angles.

Finally, the tridimensional map analysis of the electrophysiological recordings showed that there was not any correlation between the direction of the stimulus that elicited the whisking‐ neurons responses and their location within the nucleus.

### Neuroanatomical findings (Group 2)

**Figure 7:**
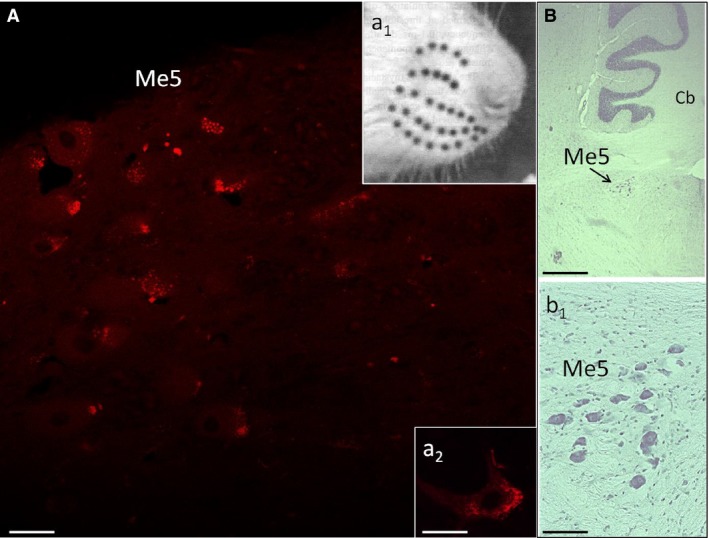
Me5 nucleus of the left‐hand side labeled by the tracer Dil injected into the ipsilateral whisker pad. (A) Fluorescence detection of the Me5 nucleus labeled by Dil. a_1_: rat muzzle showing the macrovibrissae arrangement in the whisker pad (black spots). a_2_: magnification of a Me5 labeled neuron. (B) Longitudinal histological section of the rat brain stained with cresyl violet showing the Me5 nucleus, and (b1) its zoomed view. Scale bar: 27 micron (A, a2), 250 micron (B), 150 micron (b1). Me5: trigeminal mesencephalic nucleus; Cb: cerebellum.

**Figure 8:**
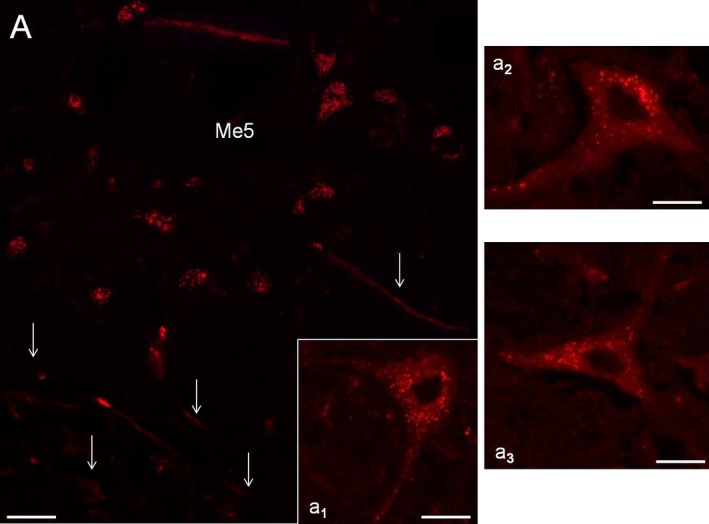
Axons and neurons of the Me5 nucleus labeled by Dil injected into the ipsilateral whisker pad. (A) Fluorescence detection of the Me5 nucleus showing axons (arrows) retrogradely labeled by the tracer injected into the ipsilateral whisker pad, and magnifications of some labeled Me5 neurons (a_1_–a_3_). Scale bar: 27 micron (A, a1, a3), 33 micron (a2).

In the animals of subgroup 2.1, the Dil injection into the whisker pad, performed to label just one vibrissae follicle‐sinus complexes, failed to identify its correspondent perikaryon in the Me5 nucleus. A similar result was found when the tracer was injected in the perifollicular area of five macrovibrissae follicles of one row (subgroup 2.2), as well as when it was injected in four macrovibrissae lying on the same arc (subgroup 2.3). In these cases, it was in fact difficult to identify labeled sets of neurons within the Me5 nucleus. On the contrary, in subgroup 2.4, in which a larger amount of the tracer was injected in the whole whisker pad, several labeled neurons were identified. They were located in the medial‐caudal part of the ipsilateral nucleus, distributed at different depths, and arranged in a single cluster of neurons organized in arrays of cellular aggregates. Their perikaryon, sizing from 30 to 55 *μ*m, was globular, oval, or triangular in shape and provided with two or multiple labeled processes (Figs. 7, 8). Results also showed that the number of these labeled Me5 neurons seemingly matched with that of the macrovibrissae (Fig. 7A,a_1_).

## Discussion

Besides the sensory afferences joining the Me5 nucleus and arising from different oral‐facial structures (Walberg 1984 for review) recent neuroanatomical and electrophysiological studies showed that the Me5 neurons are also involved in whisker pad macrovibrissae innervation (Mameli et al. 2010, 2014). With regard to the neuroanatomical representation of the macrovibrissae within the Me5 nucleus, results showed that the whisking‐neurons were distributed within the medial‐caudal part of the Me5 nucleus as a single cluster of cells with an arrangement similar to the other Me5 neurons, which, are often arranged in clusters constituted of three up to nine cells (Lazarov 2000). With regard to the uncertain results of subgroups 2.1–2.3, it is possible that the tracer amount we used was insufficient to spread inside the follicles, stain their internal structures and then the Me5 terminal axons coiled around the macrovibrissae shaft. On the other hand, the use of a larger amount of the tracer was purposely excluded because in these subgroups we wanted to be selective and avoid the whole set of macrovibrissae to be labeled, but also as a consequence of the results found in animals of subgroup 2.4. In this case, the whole whisker pad was tracer‐injected and all macrovibrissae labeled allowed us to find the correspondent fluorescent neurons in the Me5 nucleus and identify a neuroanatomical representation of macrovibrissae innervation. The whisking‐neurons were arranged in a single cluster of cells organized in an array of neurons, which shared not only the same neuroanatomical arrangement of the other sensory neurons within the Me5 nucleus, but also that of the other trigeminal neurons located in the sensory structures of the trigeminal pathway, where similar cellular aggregates are known as *barrelettes* (Zucker and Welker 1969).

With regard to the Me5 neurons innervating the macrovibrissae, results showed that they matched the features of the other common Me5 neurons involved in the sensory innervation of other oral‐facial structures. Literature data report in fact that within the Me5 nucleus coexist different neuronal populations, among which sixty percent are pseudo unipolar cells, globular or oval in shape, which are subdivided in turn into large (35–65 *μ*m) and small (up to 30 *μ*m) neurons. The remaining 40% neurons are variously sized multipolar cells (30 to 50 *μ*m) definitely considered to be real Me5 neurons (Walberg 1984; Nomura et al. 1985; Shigenaga et al. 1988a,b; Lazarov 2000 for review).

As for the functional characteristics of the Me5 whisking‐neurons, the present electrophysiological findings demonstrated that they are involved in encoding specific kinematic parameters of vibrissae deflections such as the direction, frequency, and amplitude of the applied movement. These data along with the data of our previous findings (Mameli et al. 2010, 2014) support the hypothesis of a precise functional relationship between the Me5 neurons targeting the macrovibrissae and their movements. In particular, we hypothesize that the Me5 nucleus encodes for the direction of macrovibrissae deflection using the intensities and timing of the responses elicited in its neurons as well as the spatial sequence of the neurons each time activated within the cluster. If it is so, these parameters can be sufficient for the Me5 nucleus to extrapolate the preferential direction vector along which the actual movement of the macrovibrissae is performed.

This functional hypothesis would be consistent not only with the cluster arrangement of the Me5 whisking‐neurons within the nucleus, but also with their peripheral organization at macrovibrissae level. The peripheral sensory terminals of the Me5 whisking‐neurons, coiled around the macrovibrissae shaft (Mameli et al. 2014) are in fact definitely compatible with the detection of a mechanical distortion in any direction. This could explain the reason why within the Me5 nucleus we have not found a whisking‐neurons arrangement superimposable to that showed by the geometric disposition of the macrovibrissae onto the whisker pad. Therefore, the functional activity of the whisking‐neurons is likely based on other specific information.

The present results showed that the Me5 whisking‐neurons are tuned to a preferential direction, however, given the special arrangement of their macrovibrissae receptors it is likely that each unit is activated by any direction of displacement applied to the macrovibrissae which is related to. It is in fact known that the rat can move a single macrovibrissae at time (Zucker and Welker 1969), and it is unlikely that this movement, which can be performed in any direction, does not elicit a response in the correspondent Me5 neuron. If it is so, the preferential direction of each neuron should change at any given time in accordance to the vibrissae displacement. In the present experimental protocol, the whole macrovibrissae bulk was mechanically deflected while recording the activity of individual whisking‐neurons, therefore, with regard to the neurons, only responsive to a specific direction, a possible explanation for the lack of their activation, when the deflection was performed in the non‐responsive direction, could be the induction of a surrounding inhibition phenomenon exerted by the adjacent neurons. The analysis of the single Me5 neurons responses showed that macrovibrissae deflection often induced a recruitment of previously silent Me5 whisking‐neurons. Therefore, it cannot be excluded that the Me5‐macrovibrissae signals undergo to an intra‐nuclear integration, which likely depends on both the synaptic connections operating among Me5 neurons (Lazarov 2000 for review) and the parallel activity of interneurons possibly working in network within the Me5 whisking‐neurons cluster.

The existence of a complex neural network within the Me5 nucleus has been already hypothesized in a previous study which demonstrated that the artificial whisking induced by the electrical stimulation of the buccal branch of the facial nerve, evoked polyphasic responses in the Me5 nucleus (Mameli et al. 2014). A vast literature describes a variety of excitatory and inhibitory neurotransmitters within this nucleus, among which glutamate, GABA, monoamines, histamine, and acetylcholine (Hinrichsen and Larramendi 1970; Nagy et al. 1986; Rokx et al. 1988; Copray et al. 1990; Lazarov 2000 for review). In our opinion, even the analysis of the spontaneous rhythmic whisking, frequently used by rats in their exploratory activities, supports the existence of a complex neural network within the Me5 nucleus. During this task, as reported in a previous study (Mameli et al. 2010) the Me5 neurons are alternatively excited and subsequently rhythmically inhibited in response to the ensuing macrovibrissae protraction and retraction activity. It is then possible that this fast and rhythmic change in whisking‐neurons activity is realized thanks to the complex synaptic contacts within the Me5 neural network that probably involves inhibitory interneurons. This hypothesis agrees with the literature data, which describe that in close proximity to the perikarya of the large Me5 neurons, which never showed GABA immunopositivity, there are small GABA immunoreactive cells (Lazarov 2000 for review). We speculate that they may have a role in promoting the surrounding inhibition phenomenon around the most activated Me5 neurons useful to signal to the higher CNS relay stations the actual direction the macrovibrissae were moved.

## Conclusions

The present neuroanatomical findings demonstrated that the whisker pad macrovibrissae are represented within the Me5 trigeminal nucleus by whisking‐neurons arranged in a single cluster of cells localized in its medial‐caudal part. This structural organization matches that of other trigeminal sensory neurons in the brain stem nuclei and relay stations of the trigeminal pathway (Zucker and Welker 1969).

With regard to the functional properties of these Me5 neurons, the electrophysiological data showed that they are activated by macrovibrissae deflections in specific directions and that they are also able to detect the frequency and amplitude of the executed movements. The whisking‐neurons firing rate was in fact directly correlated to the velocity and the extent of the mechanical distortion of their peripheral terminals. These data altogether seem therefore to strongly sustain a primary role of these Me5 neurons in encoding specific kinematic parameters related to macrovibrissae movements.

As for their proprioceptive function in the general contest of the brainstem trigeminal nuclei activities, it must be considered that the central terminals of the Me5 neurons extensively target the principal trigeminal nucleus (Luo et al. 1995, 2006; Wang and May 2008). This in turn receives touch information from the ipsilateral Gasser ganglion neurons, during the activation that arises from the mechanical distortion of their macrovibrissae receptors (Zucker and Welker 1969), and therefore, it may well integrate this information with the proprioceptive feedback arising from the Me5 whisking‐neurons. Moreover, as in the trigeminal ganglion, the mystacial vibrissae are somatotopically represented in relationship to their functional whisker pad architecture (Kerr and Lysak 1964; Brecht et al. 1997) the convergence of these different sensory modalities, such as touch and proprioception, may provide the trigeminal re‐transmission nuclei with a more detailed definition of the external stimuli for the higher relay stations of the trigeminal pathway.

## Conflict of Interest

All the authors declare that they have no current or potential conflict of interest including any financial support that could inappropriately influence their work.
